# A community-based exercise intervention to reduce functional risk factors for injury among stroke survivors in South Korea: a pilot randomized controlled trial

**DOI:** 10.1038/s41598-026-50927-y

**Published:** 2026-05-08

**Authors:** Dongheon Kang, Seon-Deok Eun, Jiyoung Park

**Affiliations:** 1 Department of Healthcare and Public Health Research, National Rehabilitation Center, Ministry of Health and Welfare, Seoul, 01022 Republic of Korea; 2 Department of Safety and Health, Wonkwang University, Iksan, Jeonbuk 54538 Korea

**Keywords:** Stroke rehabilitation, Community-based exercise program, Post-stroke recovery, Randomized controlled trial, Physical fitness, Health care, Medical research, Physiology

## Abstract

Stroke survivors living in the community often experience persistent impairments in muscle strength, balance, mobility, and cardiorespiratory fitness after hospital discharge, which increase vulnerability during daily activities. This pilot randomized controlled trial evaluated the feasibility of a supervised, circuit-based community exercise program and explored its potential effects on functional outcomes related to injury risk. Twenty community-dwelling adults with chronic stroke were randomized to an exercise group (EXG; *n* = 10) or a control group (CNG; *n* = 10), and 17 participants (EXG: *n* = 8; CNG: *n* = 9) completed the study. The EXG participated in a supervised multicomponent exercise program twice weekly for 8 weeks, while the CNG maintained usual activities. Outcomes included muscle strength, cardiorespiratory endurance, flexibility, balance, respiratory function, and physical performance. Between-group differences were analyzed using analysis of covariance adjusting for baseline values. The intervention showed high adherence with no adverse events. Exploratory analyses indicated improvements in multiple functional domains in the EXG compared with the CNG, although some outcomes did not reach statistical significance after adjustment. These findings support the feasibility and safety of community-based exercise and suggest potential benefits for improving functional risk factors associated with safe mobility after stroke. Larger trials are needed to confirm these results.

**Supplementary Information:**

The online version contains supplementary material available at 10.1038/s41598-026-50927-y.

## Introduction

Stroke survivors living in the community often experience persistent impairments after hospital discharge, including reduced lower-limb strength, balance control, mobility, and cardiorespiratory fitness. These functional limitations are widely recognised as modifiable factors associated with an increased risk of falls and injury-related functional decline during daily activities in community settings. Addressing such functional deficits is therefore a key component of secondary injury prevention for community-dwelling stroke survivors^1^.

Community-based exercise interventions may reduce injury risk by improving strength, balance, and mobility, thereby supporting safer participation in daily and social activities. However, many stroke survivors living in the community engage in low levels of physical activity after discharge and continue to experience functional limitations that restrict active participation in community life. Despite increasing emphasis on post-discharge community rehabilitation, evidence from randomized controlled trials remains limited regarding whether structured community-based exercise programs can meaningfully improve functional risk factors relevant to injury prevention. Therefore, the present pilot randomized controlled trial was conducted with two objectives: to evaluate the feasibility of delivering a supervised, circuit-based community exercise program to adults with chronic stroke, and to generate exploratory estimates of its potential effects on functional outcomes relevant to injury risk—including lower-limb strength, balance, mobility-related endurance, and cardiorespiratory fitness—to inform the design of a future definitive trial^2^.

Beyond their rehabilitative benefits, exercise interventions targeting strength, balance, and mobility are increasingly recognised as essential strategies for reducing injury vulnerability and promoting safe physical activity after stroke. Exercise programs are typically defined as structured, planned, and repetitive physical activities designed to improve or maintain physical fitness. In stroke populations, regular participation in exercise is strongly encouraged, as low levels of physical activity are associated with increased cardiovascular risk and functional decline, which may indirectly elevate vulnerability to injury during daily activities^3,4^.

Well-designed exercise programs that incorporate appropriate frequency, intensity, time, and type are critical for improving functional capacity after stroke. In particular, multicomponent exercise interventions combining aerobic and resistance training have demonstrated superior effects on muscle strength, cardiorespiratory fitness, balance, and mobility compared with single-modality approaches. Improvements in these functional domains are directly relevant to reducing fall risk and enhancing safe mobility in community settings. Accordingly, international clinical guidelines recommend integrating both aerobic and resistance exercises to optimise functional recovery in stroke survivors^4–7^.

Despite the established benefits of structured exercise, many stroke survivors remain insufficiently active in community settings, often participating only in low-intensity activities that provide limited functional or preventive benefit^2^. Safety concerns and the lack of structured supervision frequently limit engagement in community exercise programs, underscoring the need for supervised, standardised interventions delivered through community-based systems^2^. A recent scoping review of community-based models of mobility training after stroke highlighted that, while group exercise programs can improve walking capacity, balance, and cardiovascular function, evidence remains limited for multicomponent circuit-based programs that concurrently target multiple functional risk factors across strength, flexibility, balance, and respiratory domains^8^. In Korea, few studies have evaluated structured community-based exercise programs initiated after medical referral and delivered in real-world community environments^9^. Addressing this gap, the present pilot study builds on prior community rehabilitation initiatives to examine the feasibility of a supervised, multicomponent circuit-based exercise program—and to provide exploratory evidence of its potential effects—targeting functional risk factors relevant to secondary injury prevention in community-dwelling stroke survivors, in preparation for a future fully powered trial.

## Methods

### Participants

**Fig. 1:**
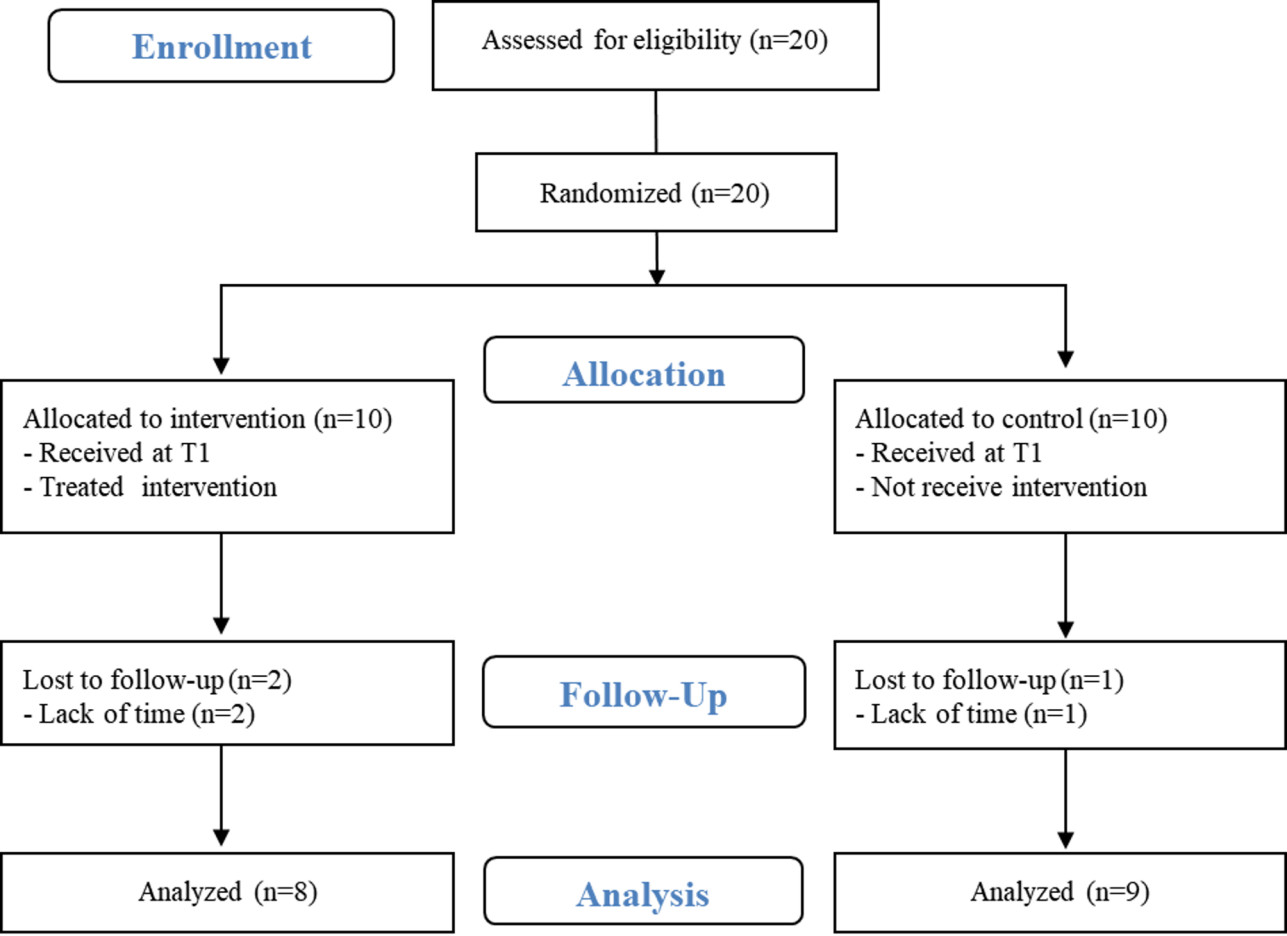
CONSORT flow diagram of participant recruitment, allocation, follow-up, and analysis. A total of 20 participants were randomized; 17 completed the study and were included in the final analysis (EXG: *n* = 8; CNG: *n* = 9). EXG: experimental exercise group; CNG: control group.

Twenty stroke patients recruited from local welfare centers, public health centers, and outpatient stroke rehabilitation centers were assessed for eligibility. Our research team, independently of the other study staff, used a computer-generated allocation schedule to randomly select participants (*n* = 20) who met the inclusion criteria before the intervention. Each participant drew a sealed envelope and was randomly assigned to either the experimental group (EXG; *n* = 10) or the control group (CNG; *n* = 10); during the intervention, two participants in the EXG and one in the CNG withdrew due to time constraints, resulting in 17 participants (EXG: *n* = 8; CNG: *n* = 9) completing all assessments and being included in the final analysis, with no missing outcome data, and the participant flow is illustrated in Fig. 1.

Stroke patients were included in the trial if they satisfied the following conditions: (a) individuals who had suffered a stroke (either ischemic or hemorrhagic), (b) individuals who had been discharged following hospitalization for stroke, and (c) individuals who provided informed consent after comprehending the details of the study. Patients were excluded from participation if they met any of the following criteria: (a) those currently hospitalized for stroke, (b) those unable to exercise due to neurological conditions unrelated to stroke (e.g., Parkinson’s disease), orthopedic limitations in the lower extremities, or other cardiopulmonary conditions, (c) those determined by the investigator to be incapable of performing the tasks, or (d) those who were pregnant.

### Study design

This trial was structured as a parallel-group, assessor-blinded, randomized pilot study involving patients who had experienced their first stroke. The interventions were administered by a certified trainer from the center, and patients with stroke were randomly assigned to an EXG or a CNG focused on activities of daily living. A researcher blinded to group allocation managed the informed consent process and data collection. The study protocol was registered (KCT0007521, registered 2022/07/19). Participants were recruited between August 2022 and November 2022, and outcomes of benefits and harms were assessed at baseline and immediately after the 8-week intervention. All participants provided written informed consent, and the study adhered to the principles outlined in the Declaration of Helsinki. The study procedures are illustrated in Fig. 1. The primary outcomes were cardiorespiratory fitness, a functional capacity related to safe ambulation in community settings, assessed by the Six-Minute Walk Test (6MWT), and lower-extremity physical performance relevant to injury prevention, assessed by the Short Physical Performance Battery (SPPB). Secondary outcomes included lower-limb muscle strength (Five Times Sit-to-Stand Test; 5STS), flexibility (Chair Sit-and-Reach Test; CSRT), balance (Berg Balance Scale; BBS), and respiratory function parameters, including forced vital capacity (FVC), forced expiratory volume in 1 s (FEV1), the FEV1/FVC ratio, peak expiratory flow (PEF), and maximal voluntary ventilation (MVV).

No formal sample size calculation was performed for this pilot RCT, consistent with recommendations for feasibility and pilot studies that are designed to assess the practicability of study procedures rather than to formally test clinical effectiveness^10^. The target enrollment of 20 participants was determined pragmatically based on the availability of community exercise facilities and the estimated eligible population within the recruitment area. This sample size was considered sufficient to assess feasibility objectives (recruitment rate, retention, adherence, and safety) and to generate preliminary effect size estimates to inform the sample size of a future definitive RCT. As a consequence, this pilot trial was not powered to detect statistically significant between-group differences in functional outcomes, and the exploratory clinical findings should be interpreted accordingly.

### Study procedure

This trial recruited stroke patients who had been discharged from the hospital. During the initial visit, the research staff informed eligible individuals about their qualifications for the study. Eligibility was determined based on specific inclusion and exclusion criteria. Participants were thoroughly briefed on the study’s purpose and procedures before providing informed consent and completing a consent form. Following this, participants completed a self-report questionnaire and underwent a face-to-face consultation with a clinician. The clinician reviewed the participants’ questionnaire responses and medical history to ensure medical suitability and safety for participation before issuing a doctor’s note, which included details such as the patient’s name, sex, age, diagnosis, date of onset, comorbidities, precautions, and medical recommendations. Before randomization, two trained evaluators measured each individual’s outcome measures. Participants were then randomly assigned to either the EXG or the CNG. Participants were advised not to reveal their group assignment to the evaluators to maintain blinding during subsequent evaluations.

### Exercise intervention

The details of the intervention program are presented in Table 1. The EXG participated in a supervised, circuit-based multicomponent exercise program consisting of 60-minute sessions held twice weekly for 8 weeks (16 sessions total). The program was structured to progressively improve overall physical fitness, incorporating the principles of specificity and progressive overload. Each session followed a circuit training format in which participants completed three rounds of four to seven exercises per round, alternating between aerobic and resistance exercises targeting upper- and lower-body muscle groups to minimize localized fatigue. Rest periods of 1–2 min were provided between sets to ensure adequate recovery.

**Table 1:** Program contents.

Phase	Category	Exercise type	Examples	Duration
Warming-up	Aerobic	Walking	Indoor Track	5 min
	Flexibility	Static/Dynamic Stretching	Whole body (e.g., Arm Swings, Hip Circles)	5 min
Main Exercise	Resistance	Lower Body	Squat, Lunge, Bridge	20 min
		Upper Body	Chest Press, Back Row, Shoulder Press, Biceps Curl, Triceps Extension	
		Trunk	Leg Raise, Reverse Crunch, Sit-up, Crunch, Back Extension, Superman Position, Deadlift	
	Aerobic	Cardiovascular	Jumping Jack, High Knee, Side-step, Front-step, Back-step, Knee-up, Pogo jump	10 min
Cooling-down	Flexibility	Static/Dynamic Stretching	Whole body (e.g., Hamstring Stretch, Shoulder Stretch)	5 min
	Cardiovascular	Walking	Slow-Paced Indoor Walking	

Prior to initiating resistance exercises, all participants completed a two-week familiarization period (Weeks 1–2) using bodyweight exercises only, to ensure safe technique acquisition before external resistance was introduced. At the outset of the program, participants also received standardized instruction in the use of the Borg Rating of Perceived Exertion (RPE) scale to ensure reliable and consistent self-reporting throughout the intervention^11^.

From Week 3 onward, resistance training was performed using TheraBands for both upper-body movements (e.g., shoulder presses, seated rows, lat pulldowns, chest presses, biceps curls, and triceps extensions) and lower-body exercises (e.g., squats, lunges, deadlifts, and bridges)^12^. Each participant’s initial resistance level was individually determined by performing lateral raises for a maximum of 15 repetitions (RMs) using the TheraBand Perceived Exertion Scale^13^. Exercises were performed in three sets of 12–15 repetitions, emphasizing quick concentric contractions, a 1-second isometric hold, and controlled eccentric phases extending beyond 2 s. For participants with restricted hand function, gloves were provided to secure the bands. Progression to a heavier resistance band from Week 6 onward was implemented when a participant was able to complete three sets of 15 repetitions with a perceived exertion consistently below 12 on the Borg scale; if perceived exertion exceeded 14 or the participant was unable to complete the prescribed repetitions, the current resistance level was maintained for an additional week before reassessment.

The aerobic component consisted of seven exercises performed without equipment (e.g., jumping jacks, sidesteps, pogo jumps), with exercise duration incrementally increased up to a maximum of 45 min over the course of the program, adjusted to each participant’s capacity.

Throughout all sessions, a target intensity of RPE 12–13 (“somewhat hard”) was consistently maintained. Heart rate was continuously monitored using a wearable heart rate monitor (Polar, Kempele, Finland) synchronized with a tablet device (iPad, Apple, Cupertino, CA, USA). The target heart rate range was set at 65–80% of each participant’s baseline peak heart rate. In cases where a participant reported an RPE of 12–13 but heart rate remained below the target range, the current exercise intensity was maintained, and the discrepancy was monitored and considered in subsequent session adjustments by the supervising trainer. Conversely, if heart rate exceeded 80% of peak heart rate regardless of reported RPE, exercise intensity was immediately reduced and an extended rest period was provided before resuming activity. These procedures ensured participant safety while accounting for the known dissociation between perceived exertion and physiological heart rate responses that may occur in stroke survivors.

The EXG intervention was administered by a certified exercise instructor with expertise in post-stroke rehabilitation. The instructor received comprehensive guidelines for managing participants in accordance with the trial protocol and was responsible for recording any adverse events occurring during the intervention.

The CNG did not participate in any targeted exercise intervention and instead maintained their usual daily routines throughout the study period. They continued to receive standard care, including medical consultations and rehabilitation services through community resources, without altering their regular lifestyle.

### Outcome measures

#### Feasibility outcomes

The primary feasibility outcomes were defined a priori in accordance with CONSORT guidance for pilot and feasibility trials^10^ and included the following four domains: (1) recruitment rate, defined as the proportion of eligible individuals who consented to participate, with a pre-specified criterion of ≥ 70%; (2) retention rate, defined as the proportion of randomized participants who completed all post-intervention assessments, with a criterion of ≥ 80%; (3) intervention adherence, defined as the proportion of prescribed exercise sessions attended by EXG participants, with a criterion of ≥ 80%; and (4) safety, defined as the incidence of adverse events (e.g., falls, musculoskeletal injuries, cardiovascular complications) during the intervention period, with a criterion of zero serious adverse events.

#### Muscular strength

The Five Times Sit-to-Stand Test (5STS) was used to evaluate lower-limb strength, a functional component associated with balance control and fall risk, in stroke patients. This test involved having the participant sit on a standard height chair (43–45 cm) with their arms crossed over their chest. The participant was then instructed to stand up and sit down five times as quickly as possible without using their arms for support. The timing began when the participant first rose from the chair and ended when they sat down for the fifth time. The test measures the time taken to complete these five repetitions, with shorter times indicating better muscle strength and functional mobility. The 5STS is recognized for its reliability and validity in assessing muscle strength, dynamic balance, and functional mobility, particularly in stroke patients. Participants were provided with one to two practice trials before the actual test to ensure accurate and consistent results. The average of three recorded measurements was used in the final analysis to account for any variability in performance. This test is valuable in clinical settings because it provides a simple yet effective measure of lower-limb strength^14^.

#### Cardiopulmonary fitness and respiratory function

The Six-Minute Walk Test (6MWT) was used to assess cardiopulmonary fitness, which is closely related to mobility and safe ambulation in community environments, in patients with stroke. This sub-maximal exercise test measures the distance a patient can walk in six minutes and is a widely recognized method for evaluating functional exercise capacity, particularly in populations with chronic conditions, such as stroke. During the test, participants were instructed to walk back and forth along a marked corridor as far as possible within the six-minute timeframe. Participants were reminded of the remaining time at regular intervals. The total distance covered in meters was recorded, serving as an indicator of the patient’s aerobic capacity. The 6MWT is highly regarded for its reliability and validity in stroke patients, reflecting their walking endurance, and has been correlated with other measures of aerobic capacity such as VO2peak. This makes it an essential tool for both clinical assessment and rehabilitation planning, providing valuable insights into a patient’s ability to perform daily activities after a stroke^15^.

Respiratory function was assessed using a digital spirometer (Pony FX, COSMED, Rome, Italy). To ensure accurate results, the examiner provided participants with a detailed explanation of the test procedures, demonstrated the process, and guided them through the measurement steps^16^. The parameters evaluated in this study included forced vital capacity (FVC), forced expiratory volume in 1 s (FEV1), FEV1/FVC, peak expiratory flow (PEF), and maximal voluntary ventilation (MVV).

#### Physical performance

The Berg Balance Scale (BBS) is a widely recognized tool for assessing balance capabilities, a key functional factor associated with fall risk in community-dwelling stroke survivors. Each activity is evaluated on a scale from 0 (inability to perform) to 4 (complete independence), with a maximum possible score of 56 points. Higher scores denote superior balance proficiency. Balance performance levels can be interpreted based on the BBS score: scores ranging from 0 to 20 points indicate a severe balance deficit, scores from 21 to 40 reflect moderate balance limitations, and scores from 41 to 56 suggest mild impairment. The scale exhibits exceptional reliability, with inter-rater reliability of 0.97 (95% CI: 0.96–0.98) and intra-rater reliability of 0.98 (95% CI: 0.97–0.99). Additionally, the smallest detectable difference on the BBS is reported to range from 2.8 to 6.6 points out of 56, highlighting the scale’s sensitivity to significant changes in balance over time^17^.

The Short Physical Performance Battery (SPPB) is a validated tool for assessing lower extremity function relevant to injury prevention during daily activities in individuals with stroke. It comprises three components: balance tests, gait speed assessment, and chair stand tests, each scored from 0 to 4, culminating in a total score ranging from 0 (worst performance) to 12 (best performance)^18^. The SPPB has demonstrated high reliability and validity in stroke populations. In clinical and research settings, the SPPB is used to evaluate physical performance, monitor functional decline, and predict outcomes, such as walking independence, in stroke patients^19^.

#### Flexibility

The Chair Sit-and-Reach Test (CSRT) is a widely used assessment tool for evaluating lower-body flexibility, with particular emphasis on hamstring and lower-back flexibility. This test is particularly relevant for individuals recovering from a stroke, as flexibility deficits can hinder mobility and functional independence, potentially increasing vulnerability during daily activities^20^.

#### Body composition

Body composition assessments were conducted before the exercise intervention. Participants’ heights were accurately measured using a stadiometer. At the same time, additional parameters, including body weight, body mass index (BMI), skeletal muscle mass, and body fat percentage, were analyzed using a bioelectrical impedance device, the InBody S10 (InBody, Seoul, South Korea). These measurements were used to describe baseline characteristics only, and changes in body composition were not evaluated as an outcome.

### Patient and public involvement

Patients and the public were not involved in the design, conduct, reporting, or dissemination plans of this research.

### Statistical analysis

Statistical analyses were conducted using SPSS version 25.0 (IBM Corp., Armonk, NY, USA), with descriptive statistics (means and standard deviations) calculated for all variables and normality assessed using the Shapiro–Wilk test, while baseline between-group differences were examined using independent samples t-tests and chi-square tests as appropriate; to evaluate between-group differences in post-intervention outcomes while accounting for baseline imbalances, analysis of covariance (ANCOVA) was performed for each outcome with post-intervention values as dependent variables, baseline values as covariates, and group allocation (EXG vs. CNG) as a fixed factor, and an additional model further adjusted for age and years since stroke onset to account for demographic and clinical heterogeneity, with within-group pre–post changes reported descriptively; statistical significance was set at α = 0.05, effect sizes were calculated using partial eta squared (ηp²) for ANCOVA (0.01 small, 0.06 medium, 0.14 large) and Cohen’s d for within-group changes (0.20–0.49 small, 0.50–0.79 moderate, ≥ 0.80 large)^21^, and no missing outcome data were observed among the 17 participants included in the final analyses (EXG: *n* = 8; CNG: *n* = 9).

### Ethics approval

This study was approved by the Institutional Review Board of National Rehabilitation Hospital (approval number: NRC2021-05-043). All procedures were conducted in accordance with the Declaration of Helsinki, and written informed consent was obtained from all participants before participation.

## Results

The results are presented with a focus on changes in functional factors related to injury risk and safe mobility among community-dwelling stroke survivors. All participants in the experimental group completed the supervised exercise sessions, and the intervention was delivered according to the planned protocol.

### Participant characteristics

The baseline characteristics of the participants, including age, height, weight, skeletal muscle mass, body fat percentage, BMI, sex distribution, stroke location, and stroke onset, are shown in Table 2. The mean age of the EXG and CNG was 51.00 ± 10.93 years and 61.44 ± 4.93 years, respectively, with a statistically significant between-group difference (*p* = 0.020), while no significant differences were observed in baseline anthropometric variables, including skeletal muscle mass, body fat percentage, and BMI; however, notable baseline imbalances were identified in the Five Times Sit-to-Stand Test (EXG: 29.66 ± 12.40 s vs. CNG: 16.08 ± 3.94 s; *p* = 0.007) and the Berg Balance Scale (EXG: 32.25 ± 14.90 vs. CNG: 49.78 ± 8.41; *p* = 0.008), and these pre-existing differences were addressed using ANCOVA to adjust for baseline values.

**Table 2:** Participants’ characteristics.

Variable	EXG	CNG	*p*
Age (years)	51.00 ± 10.93	61.44 ± 4.93	0.020
Height (cm)	168.36 ± 6.78	163.99 ± 5.52	0.163
Weight (kg)	73.97 ± 15.86	66.57 ± 10.25	0.265
BMI (kg/m²)	25.92 ± 4.25	23.58 ± 4.28	0.275
Skeletal muscle mass (kg)	30.05 ± 4.29	28.12 ± 3.73	0.338
Body fat (%)	26.07 ± 9.01	23.30 ± 5.69	0.454
Stroke onset (years)	5.47 ± 4.02	11.41 ± 6.57	0.043
FAC score	3.25 ± 1.49	3.56 ± 0.73	0.591
Sex, n (%)			
Male	8 (100.0)	8 (88.9)	-
Female	0 (0.0)	1 (11.1)	-
Stroke type, n (%)			
Hemorrhagic	6 (75.0)	7 (77.8)	-
Ischemic	2 (25.0)	2 (22.2)	-
Affected side, n (%)			
Left	5 (62.5)	6 (66.7)	-
Right	3 (37.5)	3 (33.3)	-

Values are Mean ± SD unless otherwise noted. BMI: body mass index; FAC: Functional Ambulation Classification; EXG: exercise group; CNG: control group.

### Feasibility outcomes

All four pre-specified feasibility criteria were met (Table 3). (1) Regarding recruitment rate, all 20 individuals assessed for eligibility met the inclusion criteria and were randomized (EXG: *n* = 10; CNG: *n* = 10), yielding a recruitment rate of 100%, which exceeded the pre-specified criterion of ≥ 70%; no participants were excluded following eligibility assessment, and the target enrollment was achieved within the planned recruitment period. (2) Regarding retention rate, 17 of 20 randomized participants completed all post-intervention assessments (overall: 85.0%; EXG: 8/10, 80.0%; CNG: 9/10, 90.0%), meeting the pre-specified criterion of ≥ 80%; the three withdrawals (EXG: *n* = 2; CNG: *n* = 1) were all attributable to personal reasons unrelated to the intervention or any adverse effects of the program. (3) Regarding intervention adherence, EXG completers attended a mean of 14.5 ± 1.4 of 16 prescribed sessions, with an overall session completion rate of 90.6% (116/128 sessions), which exceeded the pre-specified criterion of ≥ 80%; all eight participants individually met the ≥ 80% adherence criterion, with individual session completion rates ranging from 81.2% to 100%. (4) Regarding safety, no adverse events (falls, musculoskeletal injuries, or cardiovascular complications) were recorded throughout the intervention period, meeting the pre-specified criterion of zero serious adverse events; heart rate and Borg RPE were monitored at every session, and no participant required early termination of a session on safety grounds. These findings confirm that the program is feasible and safe to deliver in a community setting, and support progression to a future adequately powered definitive RCT.

**Table 3:** Summary of pre-specified feasibility criteria and observed results.

Feasibility domain	Pre-specified criterion	Observed result	Criterion met
Recruitment rate	≥ 70% of assessed-eligible individuals randomized	20/20 assessed were randomized (100%)	Yes
Retention rate	≥ 80% of randomized participants complete all assessments	17/20 completed (85.0%); EXG: 8/10 (80.0%); CNG: 9/10 (90.0%)	Yes
Intervention adherence	≥ 80% of prescribed sessions attended (EXG)	116/128 sessions completed (90.6%); mean 14.5 ± 1.4 sessions per participant; all 8/8 participants individually met ≥ 80%	Yes
Safety	Zero serious adverse events during 8-week program	0 adverse events recorded (falls, injuries, or cardiovascular events)	Yes

Recruitment rate: proportion of participants assessed for eligibility who were successfully randomized. Retention rate: proportion of randomized participants who completed all post-intervention outcome assessments. Intervention adherence: total sessions attended ÷ total sessions prescribed × 100%, calculated for EXG completers (*n* = 8; 8 × 16 sessions = 128 total prescribed sessions). Adverse events: falls, musculoskeletal injuries, or cardiovascular complications occurring during or immediately following supervised exercise sessions.EXG: exercise group; CNG: control group.

### Outcome measures

Results of the between-group ANCOVA and within-group descriptive changes are summarized in Table 4, showing that after adjusting for baseline values, significant between-group differences were observed in nine of ten outcomes (SPPB, 6MWT, BBS, CSRT, FVC, FEV1, FEV1/FVC, PEF, and MVV), while the Five Times Sit-to-Stand Test (5STS) did not reach statistical significance despite a clinically meaningful within-group improvement in the EXG; specifically, significant improvements favoring the EXG were identified in functional performance (SPPB: F(1,14) = 13.855, *p* = 0.002, ηp² = 0.497), cardiorespiratory endurance (6MWT: F(1,14) = 12.861, *p* = 0.003, ηp² = 0.479), balance (BBS: F(1,14) = 12.092, *p* = 0.004, ηp² = 0.463), flexibility (CSRT: F(1,14) = 6.090, *p* = 0.027, ηp² = 0.303), and all respiratory parameters (FVC: F(1,14) = 5.965, *p* = 0.028, ηp² = 0.299; FEV1: F(1,14) = 12.749, *p* = 0.003, ηp² = 0.477; FEV1/FVC: F(1,14) = 7.457, *p* = 0.016, ηp² = 0.348; PEF: F(1,14) = 17.268, *p* = 0.001, ηp² = 0.552; MVV: F(1,14) = 8.678, *p* = 0.011, ηp² = 0.383), with the EXG demonstrating consistent within-group improvements across all domains while the CNG showed minimal or no change, and no adverse events related to the intervention were reported.

**Table 4:** Between- and within-group comparisons of outcome measures (*n* = 17).

Outcome	Group	Pre (M ± SD)	Post (M ± SD)	Δ	*p* (within)	F(1,14)	*P* (ANCOVA)	ηp²
5STS (s)	EXG	29.66 ± 12.40	20.85 ± 6.19	−8.81	0.028*	0.694	0.419	0.047
	CNG	16.08 ± 3.94	17.03 ± 3.61	+ 0.95	0.008**			
SPPB (score)	EXG	5.50 ± 2.27	6.75 ± 2.25	+ 1.25	0.005**	13.855	0.002**	0.497
	CNG	7.33 ± 1.94	7.11 ± 2.03	−0.22	0.169			
6MWT (m)	EXG	213.76 ± 152.81	233.91 ± 166.05	+ 20.15	0.015*	12.861	0.003**	0.479
	CNG	233.11 ± 101.01	230.02 ± 96.59	−3.09	0.375			
CSRT (cm)	EXG	0.00 ± 6.72	4.94 ± 4.38	+ 4.94	0.001**	6.090	0.027*	0.303
	CNG	5.83 ± 9.74	5.08 ± 7.12	−0.75	0.666			
BBS (score)	EXG	32.25 ± 14.90	40.38 ± 15.61	+ 8.12	0.004**	12.092	0.004**	0.463
	CNG	49.78 ± 8.41	49.22 ± 8.47	−0.56	0.051			
FVC (L)	EXG	2.68 ± 1.51	3.12 ± 1.54	+ 0.44	0.069	5.965	0.028*	0.299
	CNG	3.06 ± 0.76	2.90 ± 0.86	−0.16	0.207			
FEV1 (L)	EXG	2.00 ± 1.32	2.70 ± 1.33	+ 0.70	0.014*	12.749	0.003**	0.477
	CNG	2.41 ± 0.55	2.20 ± 0.60	−0.21	0.098			
FEV1/FVC (%)	EXG	60.88 ± 27.54	75.00 ± 30.95	+ 14.12	0.024*	7.457	0.016*	0.348
	CNG	79.44 ± 10.69	77.56 ± 11.54	−1.89	0.364			
PEF (L/s)	EXG	3.36 ± 2.28	6.02 ± 3.37	+ 2.66	0.006**	17.268	0.001***	0.552
	CNG	4.91 ± 1.57	4.28 ± 1.19	−0.63	0.064			
MVV (L/min)	EXG	73.49 ± 58.30	93.71 ± 62.46	+ 20.22	0.125	8.678	0.011*	0.383
	CNG	81.30 ± 24.55	66.70 ± 23.18	−14.60	0.001***			

## Discussion

This study examined the effects of an 8-week community-based exercise program on functional factors associated with injury risk among individuals with stroke living in the community. The findings demonstrated that participants in the experimental group showed significant improvements in muscle strength, cardiorespiratory endurance, flexibility, balance, and overall functional performance compared with the control group. Importantly, these improvements were observed in functional domains commonly linked to fall-related vulnerability and safe mobility, suggesting potential relevance to injury risk reduction in community settings.

Lower-limb muscle strength assessed by the 5STS did not show a statistically significant between-group difference in the ANCOVA (F(1,14) = 0.694, *p* = 0.419), likely due to a substantial baseline imbalance in which the EXG had markedly worse initial performance than the CNG (29.66 ± 12.40 s vs. 16.08 ± 3.94 s; *p* = 0.007), and after adjusting for this difference, the between-group effect was no longer detectable, consistent with a regression-to-the-mean phenomenon; however, the within-group improvement observed in the EXG (Δ = −8.81 s, Cohen’s d = 0.77) exceeded the minimal clinically important difference (MCID) reported for stroke survivors,^22,23^ indicating potential clinical relevance, and future studies using stratified randomization based on baseline performance are warranted to confirm a true between-group effect.

Cardiorespiratory endurance, assessed using the 6MWT, also improved significantly following the intervention. Enhanced walking endurance is directly related to mobility capacity and safe ambulation in community settings, both of which are critical factors in reducing vulnerability during daily activities. Previous meta-analyses have reported that combined aerobic and resistance training yields greater functional benefits than single-modality exercise^7^. Taken together, these findings support multicomponent community-based exercise as a practical approach to improving mobility-related capacity and other functional risk factors relevant to safer daily activities after stroke.

Improvements in flexibility and balance, evaluated through the CSRT and BBS, respectively, underscore the importance of incorporating stretching and balance-focused training into community-based exercise programs. Reduced flexibility and impaired balance are well-recognized contributors to instability during movement, and improvements in these domains are closely linked to reduced fall risk. Consistent with previous evidence,^1^ the present findings suggest that structured exercise to address balance and flexibility may play a meaningful role in preventing secondary injuries and enhancing safe mobility among stroke survivors in community settings.

A key contribution of this study is the demonstration of the feasibility and preliminary evidence supporting the potential effectiveness of delivering structured exercise interventions in community settings. Community-based exercise programs offer a scalable and accessible alternative to hospital-based rehabilitation, reducing barriers related to cost, transportation, and facility availability. By targeting modifiable functional risk factors, such programs may serve as an accessible bridge between discharge and sustained community participation, supporting safer mobility and independence in daily life.

Several limitations should be acknowledged, including that this pilot randomized controlled trial was not designed or powered for definitive hypothesis testing and therefore provides only preliminary exploratory evidence, that despite randomization notable baseline imbalances in functional outcomes (5STS and BBS) and age were present and although adjusted using ANCOVA the small sample size (EXG: *n* = 8; CNG: *n* = 9) limits the precision of these estimates—particularly for the non-significant 5STS result which should be interpreted in light of baseline imbalance and warrants further investigation in larger stratified samples—that heterogeneity in stroke characteristics such as lesion location, time since onset, and impairment level may have contributed to variability in treatment response and limited generalizability, and that injury or fall incidence was not directly measured, making any conclusions regarding risk reduction inferential and highlighting the need for future studies incorporating prospective fall monitoring as a primary outcome.

Furthermore, the variability in stroke severity among participants may have influenced the outcomes. Stratified analyses based on stroke severity could provide a deeper understanding of how CEPs benefit different subgroups. Incorporating advanced monitoring technologies, such as wearable fitness trackers or telehealth platforms, could enhance the scalability and precision of future CEP implementations.

Future research should explore how individual cardiovascular and functional profiles interact with exercise responsiveness and injury risk after stroke. Integrating cardiovascular screening and tailored exercise prescriptions may further enhance the safety and effectiveness of community-based programs. Such approaches could contribute to the development of targeted strategies for secondary injury prevention across diverse subgroups of stroke survivors.

## Conclusion

This randomized controlled pilot trial demonstrates that a structured community-based exercise program effectively improves muscle strength, balance, mobility-related endurance, and overall physical performance among community-dwelling stroke survivors. Although injury or fall incidence was not directly assessed, the observed improvements in these functional risk factors suggest a potential contribution to safer mobility and reduced injury-related vulnerability in daily community activities after stroke.

## Supplementary Information

Below is the link to the electronic supplementary material.

Supplementary Material 1.

Supplementary Material 2.

## Data Availability

The datasets used and/or analyzed during the current study are available from the corresponding author upon reasonable request.
