# Supplementary material for: A community-based exercise intervention to reduce functional risk factors for injury among stroke survivors in South Korea: a pilot randomized controlled trial
Source: Sci Rep. 2026 May 8;16:21146. doi: 10.1038/s41598-026-50927-y (PMC13342679; doi:10.1038/s41598-026-50927-y)
Supplement: Supplementary file 1 — Supplementary Material 1. [file 41598_2026_50927_MOESM1_ESM.docx]

**RESEARCH PROTOCOL**

**Project Summary**

Stroke and spinal cord injury are major causes of long-term disability, often resulting in persistent impairments in physical function after discharge from hospital-based rehabilitation. Reduced muscle strength, balance deficits, and limited mobility can increase functional risk during daily activities, particularly in the early stages of community reintegration. However, structured rehabilitation and exercise services are often limited following discharge, highlighting the need for feasible community-based rehabilitation sports programs.

This study was designed as a pilot randomized controlled trial to evaluate the appropriateness and effectiveness of an exercise risk classification system and a structured rehabilitation sports program in individuals with stroke or spinal cord injury after hospital discharge. Participants aged 19 to 80 years who had experienced stroke or spinal cord injury for six months or longer and did not engage in regular exercise were recruited. Eligible participants were randomly allocated to an intervention group or a control group.

The intervention consisted of a supervised rehabilitation sports program tailored according to participants’ functional risk classification. The control group maintained usual daily activities without structured exercise intervention. Outcome measures focused on physical fitness and functional performance indicators relevant to safe participation in rehabilitation sports and community activities.

This pilot study aimed to generate preliminary evidence regarding the effectiveness of a structured, risk-based rehabilitation sports program and to provide foundational data for the development and application of rehabilitation sports projects in community settings. The findings were intended to inform future larger-scale studies and support the systematic implementation of rehabilitation sports programs for individuals with disabilities following hospital discharge.

**General Information**

**Protocol title**
*A Study on the Effectiveness of Exercise Risk Classification System and Program in Rehabilitation Sports Subjects: A Pilot Study*

**Protocol identifying number**

- CRIS Registration Number: **KCT0007521**
- URL: https://cris.nih.go.kr/cris/search/detailSearch.do?search_lang=E&focus=reset_12&search_page=M&pageSize=10&page=undefined&seq=25321&status=6&seq_group=22283
- Unique Protocol ID: **2021-05-043**

**Dates**

- First submitted date: 23 June 2022
- Registered date: 19 July 2022
- Last updated date: 23 July 2023

**Sponsor / Source of Monetary Support**
National Rehabilitation Center, Republic of Korea
(Project ID: **22-H-03**)

**Principal Investigator / Scientific Contact**
Seon-Deok Eun, Ph.D.
Senior Research Scientist
National Rehabilitation Center, Republic of Korea

**Public and Updating Contact Person**
Dongheon Kang, Ph.D.
Senior Researcher
National Rehabilitation Center, Republic of Korea

**Research Sites**
Community-based rehabilitation and rehabilitation sports facilities affiliated with the National Rehabilitation Center, Seoul, Republic of Korea

**Rationale and Background Information**

Individuals with stroke often experience prolonged physical impairments even after completion of inpatient rehabilitation. During the early stages of discharge, limited physical activity and insufficient access to structured rehabilitation programs can contribute to deconditioning and increased functional risk during daily activities.

Rehabilitation sports programs offer a practical approach to promoting physical activity and functional recovery in community settings. However, individuals with disabilities present heterogeneous functional abilities and medical histories, necessitating appropriate risk classification to ensure safe and effective participation. An exercise risk classification system may facilitate individualized program design and improve both safety and effectiveness of rehabilitation sports interventions.

Despite growing interest in rehabilitation sports, evidence regarding the effectiveness of structured, risk-based rehabilitation sports programs remains limited. This study was therefore designed to evaluate the appropriateness and preliminary effectiveness of an exercise risk classification system and a tailored rehabilitation sports program in individuals with stroke after hospital discharge.

**References**

References supporting the background and rationale are consistent with those cited in the main manuscript and relevant rehabilitation sports literature.

**Study Goals and Objectives**

**Study Goal**

To evaluate the appropriateness and effectiveness of an exercise risk classification system and a structured rehabilitation sports program in individuals with stroke or spinal cord injury after hospital discharge.

**Primary Objective**

- To examine changes in physical fitness and functional performance following participation in a risk-classified rehabilitation sports program.

**Secondary Objectives**

- To assess the feasibility and safety of implementing a structured rehabilitation sports program in community settings.
- To provide foundational data for future rehabilitation sports program development and application.

**Study Design**

This study was designed as a **pilot randomized controlled trial**. Eligible participants were randomly assigned to either an intervention group participating in a rehabilitation sports program or a control group maintaining usual daily activities. The study targeted community-dwelling individuals with stroke or spinal cord injury. The intervention period and outcome assessments were conducted according to a pre-specified protocol.

**Methodology**

**Participants**

**Conditions / Problems**

- Stroke (ICD-10 I00–I99; I69.4 sequelae of stroke)

**Inclusion Criteria**

- Adults aged 19–80 years
- Diagnosis of stroke or spinal cord injury for ≥6 months
- Not participating in regular exercise
- Voluntary agreement to participate after understanding the study

**Exclusion Criteria**

- Stroke duration <6 months
- Inability to exercise due to neurological, medical, or physical conditions
- Inability to participate at the discretion of investigators
- Pregnancy

**Intervention**

Participants in the intervention group participated in a structured rehabilitation sports program designed according to an exercise risk classification system. The program included supervised physical activities appropriate to participants’ functional level and medical condition. Sessions were conducted by qualified personnel at community-based facilities.

The control group continued usual daily activities without participation in the structured rehabilitation sports program.

**Procedures and Measurements**

Physical fitness and functional performance outcomes relevant to rehabilitation sports participation were assessed at baseline and after the intervention period. Standardized assessment tools and documented procedures were used.

**Randomization and Blinding**

Participants were randomly allocated to intervention or control groups. Outcome assessments were conducted by evaluators not involved in the intervention delivery.

**Flow Diagram**

A flow diagram illustrating participant recruitment, allocation, and follow-up is provided in the main manuscript.

**Safety Considerations**

Participant safety was prioritized throughout the study. Exercise sessions were supervised, and participants were monitored for adverse events. Any adverse events were documented and managed in accordance with institutional procedures.

**Follow-up**

Participants were followed for the duration of the intervention period. Follow-up procedures were in place to address adverse events if they occurred.

**Data Management and Statistical Analysis**

Data were securely stored and managed by the research team. Statistical analyses were pre-specified and focused on evaluating changes in physical fitness and functional outcomes. As a pilot study, sample size was determined based on feasibility rather than formal power calculations.

**Quality Assurance**

The study was conducted in accordance with institutional research guidelines and Good Clinical Practice principles. Standardized protocols were applied across all study procedures.

**Expected Outcomes of the Study**

This study was expected to provide preliminary evidence regarding the effectiveness and feasibility of a risk-based rehabilitation sports program for individuals with stroke or spinal cord injury. Findings were intended to inform future rehabilitation sports projects and contribute to evidence-based community rehabilitation strategies.

**Dissemination of Results and Publication Policy**

Study results will be disseminated through academic publications and presentations. Findings may also be used as foundational data for the application and expansion of rehabilitation sports programs in community settings.

**Duration of the Project**

- Recruitment and enrollment: 2022
- Intervention and assessment: 2022–2023
- Data analysis and reporting: 2023

**Problems Anticipated**

Potential challenges included participant recruitment and adherence. These issues were addressed through supervised program delivery and collaboration with community facilities.

**Project Management**

The principal investigator oversaw study design and implementation. Research staff conducted assessments and intervention delivery according to assigned responsibilities.

**Ethics**

The study was approved by the **National Rehabilitation Hospital’s Institutional Review Board**
(IRB Approval Number: **2021-05-043**, Approval Date: **12 May 2022**).
All participants provided written informed consent prior to participation.

**Informed Consent Forms**

Written informed consent forms approved by the IRB were obtained from all participants prior to study enrollment.
